# Commentary: Sensorimotor dysfunction and postural instability in older adults with type 2 diabetes mellitus: the role of proprioception and neuropathy

**DOI:** 10.3389/fnagi.2025.1658306

**Published:** 2025-09-11

**Authors:** Datian Chen, Hui Gong

**Affiliations:** ^1^Department of Geriatrics, Affiliated Haimen Hospital of Xinglin College, Nantong University, Nantong, Jiangsu, China; ^2^Department of Neurosurgery, Affiliated Haimen Hospital of Xinglin College, Nantong University, Nantong, Jiangsu, China

**Keywords:** diabetic peripheral neuropathy, proprioception, sensorimotor dysfunction, postural instability, type 2 diabetes, falls in older adults

## Introduction

Falls remain a leading cause of morbidity and mortality among older adults, especially those with Type 2 diabetes mellitus (T2DM). With over 68% of diabetic adults over 65 experiencing at least one injurious fall annually (Sun et al., 2022), there is a pressing need to identify modifiable neuromechanical contributors. Kardm et al. (2025) have made a timely and clinically meaningful contribution by highlighting proprioceptive impairments and their mechanistic ties to diabetic peripheral neuropathy (DPN). Their work builds on prior studies, such as those by Grewal et al. (2015) and Alissa et al. (2024), which identified proprioceptive deficits as critical risk factors for falls in diabetic populations, emphasizing the need for targeted sensorimotor interventions.

## Key findings and mechanistic insights

This study employed dual-digital inclinometry (DDI) and computerized dynamic posturography (CDP) to quantify proprioceptive dysfunction and postural sway. The authors demonstrated several key findings:

- **Proprioceptive error and glycemic control**: each 1% increase in HbA1c was associated with a 0.54° rise in proprioceptive error (p < 0.001), and a 1° increase predicted a 17.3% greater fall risk.
- **Stratification by neuropathy**: diabetics with TCSS scores ≥6 had 2.1 times higher sway velocity compared to non-neuropathic counterparts (d = 1.82). Mediation analysis showed that DPN accounted for 58% of HbA1c's effect on postural control (Sobel test *p* = 0.003).
- **Diagnostic thresholds**: a sway area >55 cm^2^ optimized fall prediction (Youden Index = 0.71), and a proprioceptive error ≥2.3° yielded 83% sensitivity for DPN diagnosis.

These findings are closely aligned with the American Diabetes Association's (ADA) recent call for sensorimotor screening as part of comprehensive diabetic management (American Diabetes Association Professional Practice C. 3., 2025).

## Limitations and methodological considerations

While innovative, the study has several limitations:

- **Temporal ambiguity**: longitudinal data are needed to clarify whether proprioceptive dysfunction precedes or follows microvascular complications.
- **Uncontrolled confounders**: medication effects (e.g., GLP-1 agonists, statins) and comorbid vestibular dysfunction (prevalence: ~41% in elderly diabetics) were not adjusted for (Kumar et al., 2022).
- **Measurement precision**: binary DPN classification may obscure risk gradation across TCSS 3–5. Additionally, CDP's ecological validity remains moderate (*r* = 0.32 with real-world gait variability) (Bril and Perkins, 2002).

## Future research and translational directions

### Phase 1: research priorities (0–2 years)

The 0–2-year timeframe prioritizes feasibility, focusing on interventions that leverage existing technologies and can be rapidly tested in controlled settings to establish efficacy (Table 1).

**Table 1:** Phase 1 interventions for proprioceptive dysfunction in diabetic neuropathy.

**Intervention**	**Target population**	**Outcome measures**
Vibrotactile feedback training (100–200 Hz)	TCSS 3–5	Reduction in proprioceptive error < 1.5°
VR balance perturbation	HbA1c 7–8.5%	≥15% reduction in sway velocity

### Phase 2: clinical implementation (2–5 years)

The 2–5-year timeframe allows for validation and scaling of interventions, aligning with the timeline for developing clinical guidelines and infrastructure for widespread adoption.

- **Digital screening tools**: smartphone-based DDI tools should be validated (AUC target ≥0.85), with < 3-min protocols developed for primary care use (Brognara, 2024)
- **EMR integration**: automated alerts based on combined criteria (HbA1c >7.5%, TCSS ≥3, fall history) may help stratify fall risk. Risk tiers:
  - Low: < 40 cm^2^,
  - Moderate: 40–55 cm^2^ → Physical therapy referral,
  - High: >55 cm^2^ → Multidisciplinary intervention.

## Conclusion

Kardm et al. provide robust, clinically actionable thresholds for proprioceptive screening in older adults with T2DM. These insights support the inclusion of quantitative postural assessment in diabetes care, particularly for patients with long disease duration, high HbA1c variability, or early neuropathic signs. Establishing Current Procedural Terminology (CPT) codes for proprioceptive testing and revising ADA guidelines to include routine stability evaluations could enhance both outcomes and healthcare efficiency (Zakeri et al., 2023).
